# Atypical Splicing Accompanied by Skipping Conserved Micro-Exons Produces Unique WRINKLED1, An AP2 Domain Transcription Factor in Rice Plants

**DOI:** 10.3390/plants8070207

**Published:** 2019-07-04

**Authors:** Fumiya Mano, Takuya Aoyanagi, Akiko Kozaki

**Affiliations:** Department of Biology, Shizuoka University, 836 Ohya Suruga-ku, Shizuoka 422-8529, Japan

**Keywords:** WRINKLED1 (WRI1), micro-exon, rice, *Arabidopsis*, AP2 domain transcription factor, triacylglycerol synthesis

## Abstract

WRINKLED1 (WRI1), an AP2 domain transcription factor, is a master regulator of oil synthesis in plant seeds. Its closely related proteins (WRIs) are also involved in regulating the synthesis of fatty acids, which play a role in producing oils, membranes, and other important components in plants. We found two *WRI1* genes, *OsWRI1-1* and *OsWRI1-2*, and two additional *WRI1* homologs, *OsWRI3* and *OsWRI4*, in the rice genome. *OsWRI1* was ubiquitously expressed in rice plants, including developing seeds. However, *OsWRI3* was only significantly expressed in the leaf blade and *OsWRI4* was not expressed at all. OsWRI1-1 contains amino acid sequence GCL instead of VYL, which is encoded by an independent 9-bp micro-exon that is conserved in many plant species. We found that the GCL sequence was produced by an atypical splicing accompanied by skipping of the micro-exon. Furthermore, OsWRI1-1 highly activates the transcription of the promoter for the biotin carboxyl transferase 2 gene in *Arabidopsis,* but its activity was reduced by amino acid replacement or deletion of the GCL sequence in a transient assay using *Arabidopsis* cells. Our results indicated that atypical splicing produced unique WRI1 in rice plants.

## 1. Introduction

Fatty acids are basic components of biological membranes, triacylglycerols (TAGs, the carbon storage compounds in the seeds and fruits of several plants [1]), and several other materials such as cuticular wax [2] and jasmonic acids [3]. Materials derived from fatty acids are found in several essential plant components. Therefore, it is important to properly regulate fatty acid synthesis in plants.

Fatty acids are synthesized in plastids. De novo fatty acid synthesis involves two key enzymes, acetyl-CoA carboxylase (ACCase) and fatty acid synthase (FAS) [1]. The genes for enzymes involved in fatty acid synthesis, including ACCase and FAS, and late glycolysis are shown to be regulated by WRINKLED1 (WRI1) [4]. WRI1 is a member of the APETALA2 (AP2) domain (also known as the AP2/ethylene-responsive element-binding factor (ERF) domain) transcription factors. The AP2 gene family encodes plant specific transcription factors and is divided into classes based on the number of AP2 domains that are present. One class, the AP2-like class, encodes a protein with two AP2 domains, and the other class, the ERF-like class, encodes a protein with only one AP2 domain. The AP2-like class includes AP2 [5] and AINTEGUMENTA (ANT) [6], and the ERF class includes ERFs [7], TINY [8], and ABI4 [9]. The WRIs belong to the AP2-like class and are a subclass of the ANT lineage [10].

WRI1 is thought to bind a nucleotide sequence (CnTnG(n)7CG) that is conserved among proximal upstream regions of several target genes and is called the AW-box [11]. It was thought that the main function of WRI1 was to regulate the synthesis of fatty acids during TAG synthesis. However, recent research has shown that WRI1 affects auxin homeostasis in roots [12], which indicates that WRI1 has other functions apart from the regulation of oil synthesis. In addition to WRI1, there are two additional WRI1 homologs, WRI3 (AtWRI3) and WRI4 (AtWRI4), in *Arabidopsis*. The expression patterns of these WRIs differ markedly [13] and only AtWRI1 regulates fatty acid synthesis during TAG production in seeds. AtWRI3 and AtWRI4 are required for fatty acid synthesis in other tissues [13,14]. 

Although the regulation of *WRI1* expression is still to be fully clarified, recent studies have suggested that there are several post-translational regulators in WRI1. The 26S proteasome degrades WRI1 via its interaction with the E3 adaptor BTB/POZMAT 1 (BPM1) and other BPMs [15]. Phosphorylation by KIN10 (a kinase subunit of SNF1-related protein kinase) is reported to be important during the degradation of AtWRI1 [16], whereas the interaction with 14-3-3 protein enhances the transcriptional activity and stability of AtWRI1 [17].

Structural features and functional motifs, such as the VYL [15], IDR, and PEST motifs [18], are conserved among the characterized WRI1 proteins [19]. VYL is located in the first AP2 domain (responsible for DNA binding) and is encoded by an independent 9-bp micro-exon in *Arabidopsis* [20] and castor bean (*Ricinus communis*) [21]. In *Arabidopsis,* site-directed mutagenesis of amino acids within VYL failed to restore the full oil contents of *wri1-1* seeds, which indicated that the VYL sequence is required for AtWRI1 function. There are splice variants of castor bean WRIs (RcWRI1). RcWRI1-A contains VYL, but RcWRI1-B lacks VYL [21]. In contrast to AtWRI1, both RcWRI1s restore the full oil content of *wri1-1*, even though RcWRI1-B lacks VYL. These contradictory results have raised questions about whether or not the VYL sequence has important effects on WRI1 activity.

We found that rice (*Oryza sativa*) WRI1 (OsWRI1) contains a GCL sequence instead of VYL, although there is a trace of the micro-exon encoding VYL in the rice genome. The results also show that OsWRI1 has high transcriptional activity in a transient assay using cultured *Arabidopsis* cells. Our data indicated that the original micro-exon was skipped in the *OsWRI1* gene and that atypical splicing had occurred in the past, which led to the high performance of OsWRI1. The atypical splicing had probably been fixed over the course of evolution. 

## 2. Materials and Methods

### 2.1. Sequence Analysis

The amino acid sequence of AtWRI1 was obtained from TAIR10 (https://www.arabidopsis.org/) and the sequence was used as the query for a BLAST search (TBLASTN) against the National Center for Biotechnology Information (NCBI, https://blast.ncbi.nlm.nih.gov/Blast.cgi). We collected the sequences of WRI1 homologs of the Poaceae family: rice (*Oryza sativa*), solghum (*Solghum bicolor*), *Setaria italican, Brachypodium distachyon,* and *Brachypodium stacei.* We also obtained the sequences of AtWRI2-4 from TAIR10 and other WRI1s (RcWRI1, CoWRI1, EgWRI1, SlWRI1, and ZmWRI1s) which had already been identified as WRI1 [21,22,23,24] from the NCBI data base. All reserved amino acid sequences (full-length) were subjected to Clustal-W 2.1 (DNA Data Bank of Japan: http://clustalw.ddbj.nig.ac.jp/index.php?lang=ja) and a phylogenetic tree was generated. As AtWRI, AtWRI2-4, and ZmWRI1s were classified into different clades, the sequences that classified to these three clades were reserved as WRI1 and WRI1-like proteins. At this point, we removed rice AP2 domain proteins found in the first BLAST search (Os03g56050, Os04g55970, Os01g67410, Os04g42570, and Os02g40070) from the rice WRI1 or WRI1-like candidates because they were out of clades of WRI1 or WRI1-like proteins. Thereafter, the reserved sequences were subjected to Clustal-W again and the final phylogenetic tree was generated. Multiple sequence alignment in Figure 3 was performed using Clustal-W mentioned above. Full length sequences of WRI1 proteins from various species were aligned using Clustal-W and sequences surrounding VYL were shown. 

### 2.2. Expression Analysis

Rice plants (*Oryza sativa* L. cv. Nipponbare) were grown in soil under greenhouse conditions. Total RNA was extracted from seeds at 12 days after flowering (DAF), 3-day-old seedlings, shoots and roots from 1-week-old seedlings (1w), leaf blades from 2-week-old (2w) or 2-month-old (2m plants, and panicles measuring 2, 10, and 20 cm length. 

Total RNA was extracted using ISOGEN (Nippon Gene, Toyama, Japan) in accordance with the manufacturer’s protocol. After DNase treatment (Takara, Ohtsu, Japan), first-strand cDNA was synthesized using 1 μg of total RNA, oligo (dT), and ReverTra Ace (Toyobo, Osaka, Japan). 

Real-time PCR was carried out with SYBR premix EX Taq II (Takara) using the LightCycler 480 System II (Roche, Mannheim, Germany). Gene expression levels relative to the *OsUBQ1* reference gene were calculated by the ΔΔCt method [25]. The experiment was performed in triplicate for each gene, including the no-template and no-reverse-transcriptase controls. The primer sets used for the expression analysis are shown in Table S1. 

### 2.3. Vector Construction

Effector vectors were constructed by introducing *OsWRI1* or *AtWRI1* sequences downstream of the CaMV 35S promoter. The open reading frames (ORFs) for *OsWRI1* and *AtWRI1* were amplified by PCR using the primer sets shown in Table S1. The full-length ORF coding sequences for *OsWRI1* and *AtWRI1* were amplified using OsWRI1 XbaIF/OsWRI1 KpnIR, and AtWRI1 XbaIF/AtWRI1 KpnIR, respectively. Mutations were introduced using a primer extension method. For example, to make OsWRI1 GCQ, two PCR fragments were amplified using primer sets OsWRI1 XbaIF/OsWRI1 GCQR and OsWRI1 GCQF/OsWRI1 KpnIR. The two amplified fragments were mixed and used as templates in a final PCR reaction to amplify the full-length *OsWRI1*.

The reporter vector *BCCP2:LUC* was supplied by K. Nakamura (Nagoya University, Japan). The 1.2 kb sequence upstream of the ATG codon in the AtBCCP2 gene was introduced upstream of the luciferase reporter gene (*LUC*) in pBI221 [11]. 

### 2.4. Transient Assay

Transient assays were performed as described previously [26]. A suspension of protoplasts (150 µL; 10^7^ protoplasts mL^−1^) prepared from Arbidopsis T87 cell cultures was co-transfected with 10 µg each of the *LUC* reporter and the effector plasmid DNAs, and 5 µg of 35S:hRLUC were used as the internal control plasmid. After incubation at 22 °C for 20 h, the protoplasts were collected and reporter activities were measured. The LUC and hRLUC activities were measured using the Dual-Luciferase Reporter Assay system (Promega, Madison, WI, USA). The LUC activity was normalized according to the hRLUC activity in each assay, and the relative ratio was determined by comparing normalized ratio with that obtained from the empty vector. Three or four independent measurements of LUC activities were averaged and statistically analyzed by a Student’s *t* test.

## 3. Results

### 3.1. Molecular Characterization of WRI1 like Transcription Factors in Rice 

**Figure 1:**
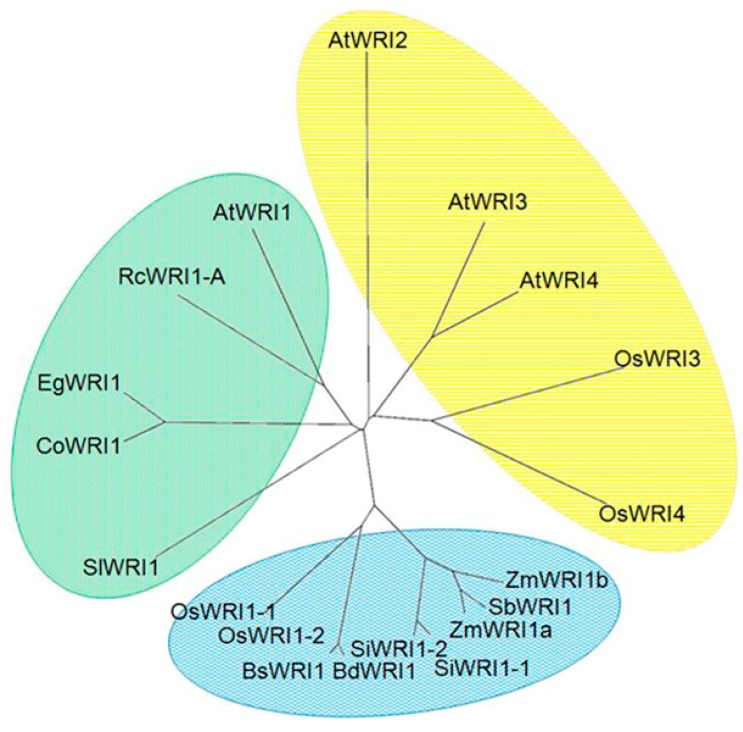
Phylogenic analysis of WRINKLED1 (WRI1)-like proteins. The tree was generated using amino acid sequences of WRI1 like proteins from different species. These were rice (*Oryza sativa*) (Poaceae): OsWRI1-4; *Arabidopsis* (Brassicaceae): AtWRI1-4; maize (Poaceae): ZmWRI1a and ZmWRI1b (Pouvreau et al. 2011); solghum (*Solghum bicolor*) (Poaceae): SbWRI1 (XP 020406865); *Setaria italica* (Poaceae): SiWRI1 1 (XP 004977441) and SiWRI1 2 (XP 004978469); *Brachypodium distachyon* (Poaceae): BdWRI1 (XP 003578997); *Brachypodium stacei* (Poaceae): BsWRI1; tomato (*Solanum lycopersicum*) (Solanaceae): SlWRI1 (XP 004231231); coconut (*Cocos nucifera*) (Arecaceae): CoWRI1 (AFH68065); oil palm (*Elaeis guineensis*) (Arecaceae): EgWRI1 (XP 010922928); and castor bean (*Ricinus communis*) (Euphorbiaceae): RcWRI1-A and RcWRI1-B (Ji et al. 2018). The phylogenetic tree was illustrated using Tree View from a tree file produced by CLUSTAL-W.

An analysis of the GenBank database showed that four genes encoded WRI1 like protein in the rice genome (*OsWRI1-1*: Os11g03540, *OsWRI1-2*: Os12g03290, *OsWRI3*: Os5g45954, and *OsWRI4*:Os6g05340). *OsWRI1-1* and *OsWRI1-2* are located in positions where genome duplication has occurred [27]. Therefore, the sequences of these genes showed high homology including introns and the 5′ and 3′ untranslated regions (UTRs)(Figure S1). We collected the WRI1 like sequences from several plants, including four *Arabidopsis* WRI1 like proteins, AtWRI1-4, to clarify the relationship between these four rice WRI1 like proteins and WRI1 proteins from other plants. The phylogenic analysis (Figure 1) indicated OsWRI3 and OsWRI4 are closer to AtWRI2, AtWRI3, and AtWRI4. Furthermore, the phylogenic tree showed that WRI1s from Poaceae are not in the same clade as those from *Arabidopsis*, coconut (*Cocos nucifera*) (CoWRI1), oil palm (*Elaeis guineensis*)(EgWRI1), tomato (*Solanum lycopersicum*) (SlWRI1), and castor bean (RcWRI1-A and RcWRI1-B). The OsWRI1-1 and OsWRI1-2 are in the same clade as maize (*Zea mays*) WRI1s (ZmWRI1a and ZmWRI1b), which have been identified as WRI1 orthologs, respectively [24]. Therefore, we assumed that OsWRI1-1 and OsWRI1-2 are WRI1 orthologs and OsWRI3 and OsWRI4 are orthologs of AtWRI3 and AtWRI4.

The expressions of WRI1 like genes in rice were also analyzed by quantitative RT-PCR (qRT-PCR). The *OsWRI1-1* and *OsWRI1-2* sequences were almost identical (Figure S1), including the 5′- and 3′-UTRs, which meant that we could not design primers to distinguish them. Therefore, we analyzed the expression of both *OsWRI1* genes without distinction between *OsWRI1-1* and *OsWRI1-2.*

**Figure 2:**
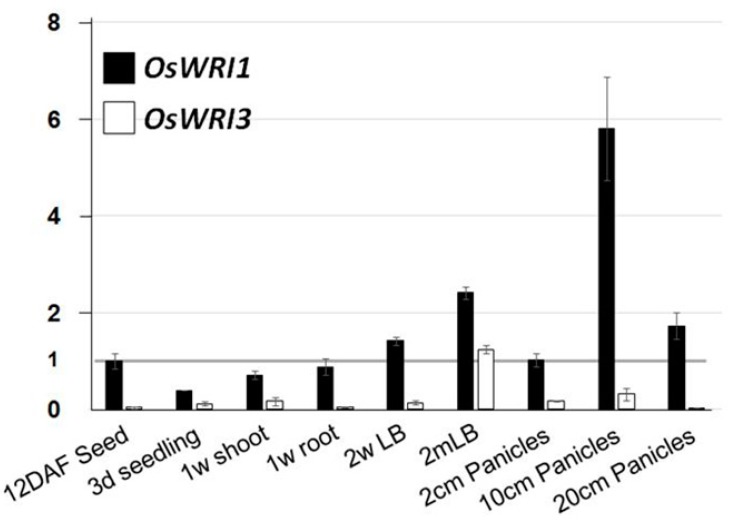
Expressions of *OsWRI* genes in various rice organs. The expression profiles of OsWRI1 and OsWRI3 were established by qRT-PCR in various organs of rice. The gene expression levels were normalized against the expression of *OsUVQ1*. Relative expression levels are presented as the ratio of the expression level of *OsWRI1* in 12 DAF seeds. The error bar represents SD. DAF: days after flowering; LB: leaf blade; d: day; w: week; m: month.

The *OsWRI1* gene was expressed in all tissues analyzed and high expression was detected in the panicle (10 cm) and leaf blade (2 months) (Figure 2). The highest level of *OsWRI3* expression was detected in the leaf blade (2 months), while the expression was low in other tissues. *WRI4* expression was not detected in all tissues examined by either semi-quantitative RT-PCR, using the two different pairs of primers listed in Table S1, or qRT-PCR. Therefore, the result of *OsWRI4* was not shown in Figure 2. The result that only *OsWRI1* was expressed in developing seeds indicated that *OsWRI1* is involved in oil synthesis in the rice embryo similar to *AtWRI1*.

### 3.2. Genome Structure

**Figure 3:**
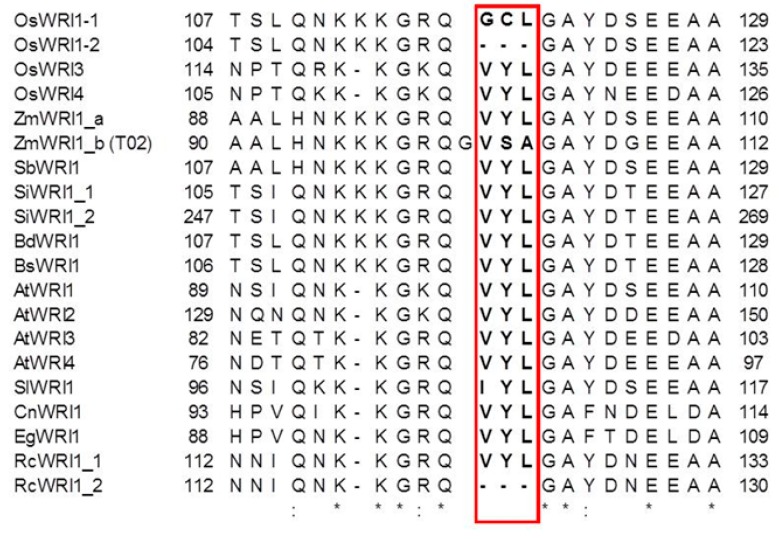
Alignment of sequences around VYL in WRI1s from various species. VYL and the corresponding regions are boxed. Amino acid sequences for the WRIs in rice (Poaceae): OsWRI1-4; *Arabidopsis* (Brassicaceae): AtWRI1-4; maize: ZmWRI1a and ZmWRI1b (Tang et al. 2019); *Solghum bicolor* (Poaceae): SbWRI1 (XP 020406865); *Setaria italica* (Poaceae): SiWRI1 1 (XP004977441) and SiWRI1 2 (XP 004978469); *Brachypodium distachyon* (Poaceae): BdWRI1 (XP 003578997); *Brachypodium stacei* (Poaceae): BsWRI1; tomato: SlWRI1 (XP 004231231); coconut (Arecaceae): CoWRI1 (xxxAFH68065); oil palm (Arecaceae): EgWRI1 (XP 010922928); and castor bean (Euphorbiaceae): RcWRI1-A and RcWRI1-B (Ji et al. 2018). The numbers on each side indicate the amino acid number from the N-terminal. The asterisk and colon symbols correspond to residue identity and similarity, respectively.

WRI1 orthologs from many diverse plant species have been reported to contain the conserved “VYL” amino acids, which affect WRI1 activity, and the nine nucleotides encoding VYL form an independent micro-exon [20,21,28,29]. The sequence alignments for OsWRI1s and other WRI1 proteins show that OsWRI1-1 contains another amino acid sequence, GCL, instead of VYL, and that WRI1-2 lacks the VYL sequence, whereas OsWRI3 and OsWRI4 contain VYL (Figure 3). No other reported WRI1s apart from OsWRI1-1 contain GCL instead of VYL (Figure 3) [20,29]. 

**Figure 4:**
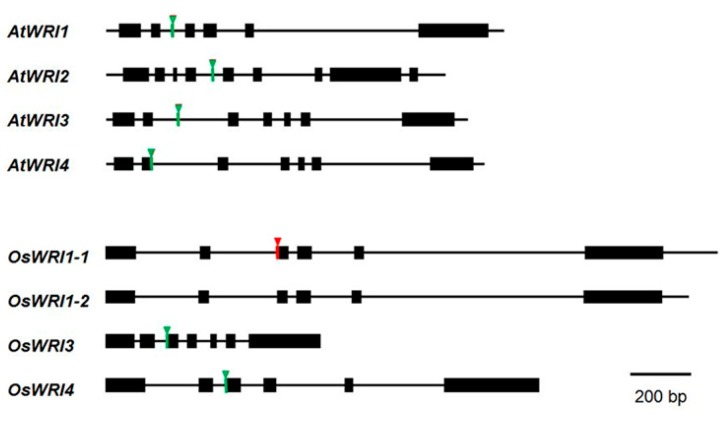
Comparison of the gene structures for *WRI1* like genes from rice and *Arabidopsis.* Schematic comparison of the gene structures for WRI genes from rice and *Arabidopsis*. Black boxes and the bold lines show the exon and intron regions, respectively. The bars with inverted triangles indicate the position of a sequence encoding VYL (in green) or GCL (in red).

We then analyzed the genome structures of the *OsWRIs* (Figure 4). Interestingly, the nine nucleotide sequences corresponding to the GCL or VYL sequences in all of the *OsWRI1*-like genes are not on the independent exon. Instead, with the exception of OsWRI1-2, the sequences are found on the 5′ end of the third exon. In *Arabidopsis*, only *AtWRI4* does not have a micro-exon encoding VYL. Instead, the sequence for VYL is on the 3′ end of the second exon (Figure 4). The exon-intron structures are almost conserved among *OsWRIs*, except that the last exon of *OsWRI3* is divided in two exons. The *WRI1* gene structures for the species in the phylogenic tree shown in Figure 1 were analyzed. The VYL sequence in all of the genes analyzed had an independent third micro-exon (Figure S2)

**Figure 5:**
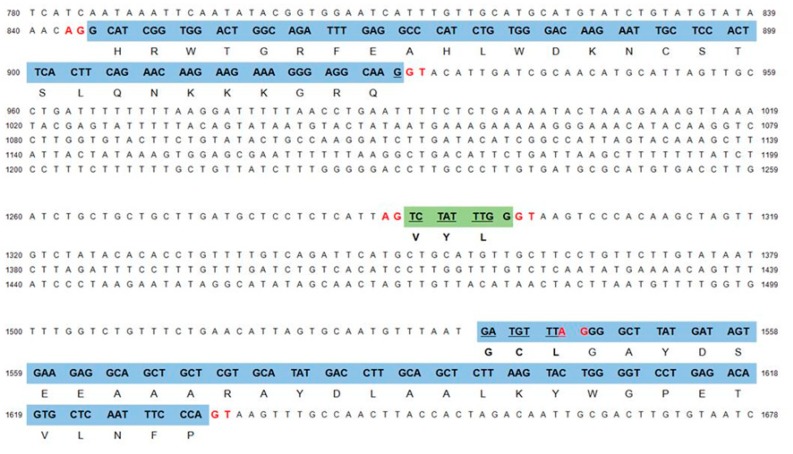
Trace of the micro-exon for VYL in the *OsWRI1-1* gene. The shaded regions are exons. The nucleotides involved in the GT-AG rule are red and in bold. The numbers at each side indicate the nucleotide number from the translation initiation site (ATG).

OsWRI1-1 contains GCL instead of VYL. Therefore, we searched for a trace of the micro-exon encoding VYL in the second intron of the *OsWRI1-1* gene. We found the sequence encoding VYL, which was sandwiched between the AG and GT sequences (Figure 5). We also found the AG sequence in the last nucleotide of the sequence encoding GCL. The AGTCTATTTGGGT sequence, which carries the sequence corresponding to VYL, can be the micro-exon, and the sequence corresponding to GCL can be spliced out when the GT-AG rule is applied (Figure 5). This result suggested that *OsWRI1-1* originally contained the VYL sequence, and that VYL was lost by atypical splicing and was replaced by GCL.

### 3.3. The Activities of OsWRI1-1 and Its Mutant.

In *Arabidopsis*, replacement of amino acids within the VYL sequence in AtWRI1 reduced or eliminated its function (Ma et al. 2013). However, in castor bean, deleting VYL did not affect RcWRI1-B function [21]. These contradictory results mean that it is not clear whether OsWRI1 is a regulatory transcription factor during oil biosynthesis. To evaluate the transcriptional activity of OsWRI1s, we tried to isolate full length of OsWRI1-1 and OsWRI1-2. We cloned several OsWRI1 genes by RT-PCR using cDNA derived from rice seeds (12 days after flowering) and their sequences were analyzed. All the clones analyzed contained the OsWRI1-1 sequence, but we could not obtain the OsWRI1-2 clone. As the amino acid sequences of OsWRI1-1 and OsWRI1-2 are 98% identical (Figure S1a), we could make OsWRI1-2 by deletion of GCL sequence from OsWRI1-1. Therefore, we used the cloned WRI1-1 for the experiments hereafter.

The transcriptional activity of OsWRI1-1 and its mutant were measured by transient assay using cultured *Arabidopsis* cells to determine OsWRI-1 transcriptional activity levels and investigate whether the mutagenesis of amino acids within the GCL affects activity.

We made several mutant OsWRI1-1s. These were OsWRI1-1 containing VYL (OsWRI1 VYL), GCQ (OsWRI1 GCQ) instead of GCL, and OsWRI1-1 lacking GCL (OsWRI1ΔGCL). The genes for these mutant OsWRI1s and the original OsWRI1-1 were placed under the control of the cauliflower mosaic virus (CaMV) 35S promoter so that effector vectors could be constructed. To compare their activities, we also prepared the effector vectors for AtWRI1 and its mutants, which were AtWRI1 containing GCL instead of VYL (AtWRI1 GCL) and AtWRI1 lacking VYL (AtWRI1ΔVYL), which could then be used as comparisons. 

The promoter of the *Arabidopsis BCCP2* gene was used as a reporter. The *BCCP* gene encodes biotin carboxyl carrier protein, which is one of the subunits of acetyl-CoA carboxylase (ACCase). *Arabidopsis BCCP2* shows seed specific expression and is a major target of WRI1. The reporter vector was constructed by ligating an approximately 300 bp upstream region from the ATG of the *Arabidopsis BCCP2* gene to a region that was upstream of the luciferase (LUC) gene (BCCP2:LUC). The fragment consisted of 122 bp of the 5′-UTR sequence that contained two AW boxes [11,30]. 

**Figure 6:**
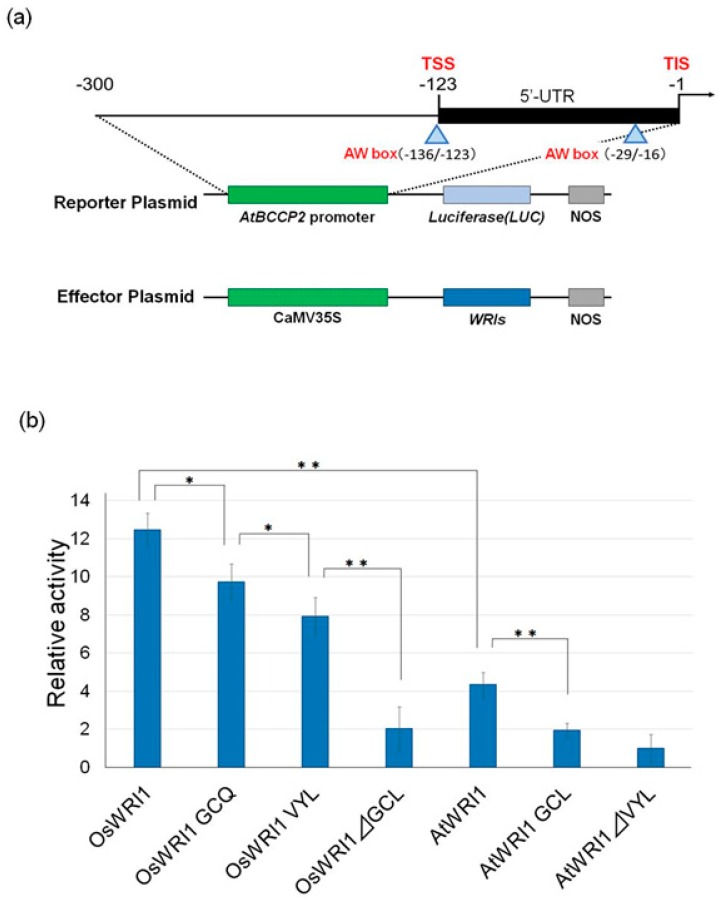
Transcriptional activities of OsWRI1-1 and AtWRI1 and their mutants. (**a**) Schematic diagram of the reporter and effector plasmids used in transient assays. TSS: Transcriptional start site. TIS: Translation initiation site. (**b**) Transient assays to determine the transcriptional activities. The transcriptional activities of the proteins were measured by transient expression assay using cultured *Arabidopsis* cells. An empty vector was used as a control and all LUC activities are expressed relative to this control (value set at 1). Values shown are the average results from three or four independent experiments. The error bars represent SD. Asterisks denote significant difference levels (p* < 0.05, p** < 0.01).

When OsWRI1 was used as an effector, the relative activity was approximately 12 (Figure 6). When GCL was replaced by GCQ (OsWRI1 GCQ) or VYL (OsWRI1 VYL), the OsWRI1 activity decreased slightly, and when GCL was deleted (OsWRI1 ΔGCL), activity decreased considerably, but weak activity was still detected. In contrast, AtWRI1 activity was much lower than OsWRI1 (approximately one third of OsWRI1-1). When VYL was replaced by GCL (AtWRI1 GCL), its activity was reduced to levels that were similar to OsWRI1 ΔGCL, and activity was completely lost when VYL was deleted (AtWRI1ΔVYL) (Figure 6). 

## 4. Discussion

In rice plants, there are four *WRI1* like genes, but only *OsWRI1* is expressed in developing seeds. Therefore, *OsWRI1* is considered to be involved in seed fatty acid synthesis. OsWRI3 and OsWRI4 are closer to AtWRI2, AtWRI3, and AtWRI4 than AtWRI1 (Figure 1). *AtWRI3* and *AWRI4* are reported to be involved in cutin synthesis in floral organs and stems in *Arabidopsis* [13,14]. *OsWRI3* was expressed mainly in the 2-month-old leaf blade (Figure 2). *OsWRI3* is probably required for cuticular synthesis in rice leaves. *OsWRI4* was not detected in any of the tissues examined. However, although *OsWRI4* expression was not detected in our experiments, it might be expressed in other specific tissues. In contrast, *OsWRI1* was expressed in all the tissues examined (Figure 2). These results indicated that *OsWRI1* has functions in several tissues besides seeds. In maize plants, there are two *WRI1* genes, *ZmWRI1a* and *ZmWRIb*. Although both of them showed strongest expression in young maize kernels, they were expressed to various extents in all organs [24]. The results indicate that maize *WRI1* genes function in several organs, different from *AtWRI1*. *ZmWRI1a* and *ZmWRI1b* showed slightly different expression patterns, indicating that they have different physiological roles [24]. Rice plants also contain two *WRI1* genes; however, different from the maize WRI1 genes, the nucleotide sequences of both the *OsWRI1* genes show extremely high homology, including their introns and 3′- and 5′ -UTR (Figure S1). There are two sequences (9 bases) which are specific to *OsWRI1-1* (Figure S1b). Therefore, we tried to design specific primers for *OsWIR1-1* using these sequences. However, we could not amplify the fragment for some unknown reason although we tried PCR under various conditions (data not shown). Therefore, we could not distinguish the expression of *OsWRI1* genes.

Alignment of WRI1 sequences from rice, *Arabidopsis*, and other species showed that the VYL sequence was replaced by GCL in OsWRI1-1. The VYL sequence is reported to be encoded by an independent exon, and site-directed mutagenesis within the VYL sequence in AtWRI1 failed to restore the oil content in the *wri1* mutant [20]. The WRI1s from most species whose sequences are available contain VYL, but some WRI1s contain IYL instead of VYL (Figure 3) [20]. IYL is thought to be produced as a replacement for the first nucleotide in the codon for V (G to A). The first amino acid “V” seems less important among VYLs [20,31] and can be replaced. In contrast, the GCL sequence cannot be produced by replacing one nucleotide. An analysis of the *OsWRI1-1* genome sequence showed that GCL was not encoded by an independent micro-exon, but was located in the third exon (Figure 4). Furthermore, a trace of the independent micro-exon encoding VYL was found in the intron between the second and third exons. The micro-exon followed the GT-AG rule, whereas the 5′ site adjacent to the start of the third exon did not follow the GT-AG rule (Figure 5). Recently, Tang et al. (2019) reported that ZmWRI1b contains VSA instead of VYL. The maize GDB (https://www.maizegdb.org/) shows that there are five transcript variants of *ZmWRI1b* and the canonical transcripts (T02) among them contain VSA. We searched for a trace of the sequence coding VYL in the second intron of *ZmWRI1b* (T02) and found the sequence coding VYL, although the sequence was sandwiched between AG and GC, not between AG and GT (data not shown). The results indicated that the process of VSA sequence generation in ZmWRI1b was similar to that of GCL sequences in OsWRI1-1. In addition, the atypical splicing in ZmWRI1b produced additional G’s at the N terminal of VSA (Figure 3).

There was no independent micro-exon in the *OsWRI3* and *OsWRI4* genome sequences. The sequences encoding VYL were located in the 5′ site of the third exon (Figure 4). In contrast to *OsWRI1-1*, there was no trace of the micro-exon in the introns of the *OsWRI3* and *OsWRI4* genes (data not shown), which indicated that *OsWRI3* and *OsWRI4* had simply lost the introns between the micro-exon and the current third exon. It is presumed that the intron loss occurred before *OsWRI3* and *OsWRI4* split from a common ancestor.

In the transient assay that used BCCP2:LUC as a reporter construct (Figure 6), OsWRI1-1 showed much higher activity than AtWRI1. Replacing GCL in OsWRI1-1 with GCQ or VYL slightly reduced activity, although their activity was still higher than that of AtWRI1 (Figure 6). However, deleting GCL remarkably reduced OsWRI1-1 activity to its lowest level. In contrast, replacing VYL in AtWRI1 with GCL reduced its activity to levels that were similar to OsWRI1ΔGCL, but deleting VYL abolished all activity. The results indicated that GCL is a better sequence for OsWRI1 than VYL, whereas VYL is better for AtWRI1. Although OsWRI1 activity was not severely affected when VYL was replaced by GCL, deleting GCL (OsWRI1 ΔGCL) had a severe effect, which was similar to AtWRI1. Both OsWRI1 and AtWRI1 almost lost their activity when GCL or VYL, respectively, were deleted, which suggests that the sequences are important for their activity. The results were different from that of castor WRI1 [21]. 

The OsWRI1ΔGCL sequence was almost the same as OsWRI1-2, which indicated that the transcriptional activity of OsWRI1-2 is not high. The OsWRI1-2 sequence was derived from the data base and it is not known whether the annotation is correct or whether it is expressed because we could not isolate OsWRI1-2. We don’t know whether *OsWRI1-2* is functional or not at this time.

We could not examine the binding activity of OsWRI1-1 and its mutant to the AW box because we failed to express recombinant OsWRI1, which meant that an electrophoresis mobility shift assay (EMSA) could not be undertaken. Therefore we could not determine whether the reduced transcriptional activity shown by OsWRI1-1 after mutagenesis or GCL deletion was due to a reduction in binding activity. Krizek (2003) reported that site-directed mutagenesis of VYL in *Arabidopsis* AINTEGUMENTA (ANT) reduced or abolished the DNA binding activity to the ANT binding sequence [31], which indicated that VYL plays an important role in the DNA binding of ANT. It is possible that the DNA binding activity was much reduced or lost after GCL or VYL deletion in OsWRI1-1 and AtWRI1, respectively. 

Although the VYL sequence is well conserved among the AP2-like proteins and has an important role in the function of some proteins (AtWRI1, OsWRI1, and ANT) (Figure 6) [20,31], there are several AP2-like proteins that lack the VYL sequence [10,20,29]. Furthermore, the deletion of the VYL sequence does not affect the function of some proteins [21]. It is not clear why the importance of the sequence VYL varies in different proteins.

In summary, our results suggest that OsWRI1 originally contained VYL, which was encoded by an independent micro-exon and that atypical splice-out occurred by chance. This led to the production of an OsWRI1 containing GCL instead of VYL. The OsWRI1 containing GCL was a high-performance transcription factor and the atypical spicing was subsequently fixed by an unknown mechanism. Furthermore, *OsWRI1* expressed in most tissues and has functional roles in all parts of the rice plant.

## Supplementary Materials

The following are available online at https://www.mdpi.com/2223-7747/8/7/207/s1, Figure S1: (a) Comparison of amino acid sequence of OsWRI1-1 and OsWRI1-2. (b) Comparison of CDS sequence of OsWRI1-1 and OsWRI1-2. (c) Comparison of genome sequence of OsWRI1-1 and OsWRI1-2., Figure S2: Comparison of gene structure of WRI1 like genes from several plants. Table S1: Primers used in this study.
